# Synergistic antibacterial photocatalytic and photothermal properties over bowl-shaped TiO_2_ nanostructures on Ti-19Zr-10Nb-1Fe alloy

**DOI:** 10.1093/rb/rbac025

**Published:** 2022-05-04

**Authors:** Yan Wu, Zichao Deng, Xueying Wang, Aihua Chen, Yan Li

**Affiliations:** 1 School of Materials Science and Engineering, Beihang University, Beijing 100191, China; 2 Beihang Hangzhou Innovation Institute Yuhang, Beihang University, Hangzhou 310023, China; 3 Biomaterials Laboratory of the Medical Device Inspection Institute, National Institutes for Food and Drug Control, Beijing 102629, China; 4 Beijing Advanced Innovation Centre for Biomedical Engineering, Beihang University, Beijing 100191, China

**Keywords:** bowl-shape TiO_2_, Ti-19Zr-10Nb-1Fe alloy, antibacterial, photocatalytic, photothermal

## Abstract

As implant substitutes are increasingly applied to the clinic, the infection caused by implants has become one of the most common complications, and the modification of the antibacterial function of the implant can reduce such complications. In this work, a well-defined bowl-shaped nanostructure coating with photocatalytic and photothermal synergistic antibacterial properties was prepared on Ti-19Zr-10Nb-1Fe (TZNF) alloy. The coating is obtained by spin-coating and sintering TiO_2_ precursors templated from self-assembled microspheres of polystyrene-poly(4-vinylpyridine) (PS-P4VP) amphiphilic block polymer on TZNF alloy. PS-P4VP provides the bowl-shaped TiO_2_ nanostructures doped with C, N elements, reducing the band-gap of TiO_2_, which can absorb near-infrared (NIR) light to release reactive oxygen species and produce photothermal conversion. The bowl structure is expected to enhance the utilization of light via the reflection in the confined space. The bowl-shaped surface has 100% antibacterial rates after 30 min of NIR light irradiation. In addition to antibacterial properties, the bowl-shaped surface has better hydrophilicity and protein adsorption capacity. The amount of protein adsorbed on TZNF with the bowl-shaped structures was six times that of TZNF. Hence, the bowl-shaped nanostructure can promote the proliferation and adhesion of osteoblasts, the cell proliferation rate was increased by 10–30%.

## Graphical Abstract

**Figure:**
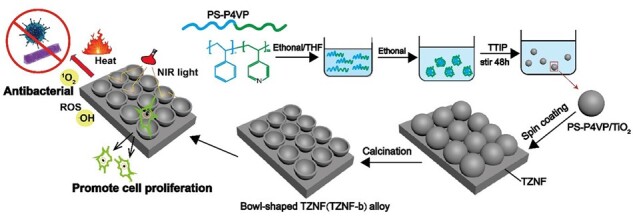

## Introduction

Titanium alloy is one of the most commonly used materials for orthopedic implants because of its favorable mechanical properties and biocompatibility. Conventional titanium alloys commonly used in biomedicine are Ti-6Al-4V and Ti-6Al-7Nb, etc., but their applied Young’s modulus is higher than that of bones and are prone to release harmful ions, causing pressure shielding and toxic side effects [1, 2]. Based on traditional titanium alloy implants, a series of titanium alloys made of low elastic modulus and non-toxic elements have been developed, such as Ti-Zr [3], Ti-Al [4], Ti-13Nb-13Zr [5], etc.

When titanium alloy is implanted, although strict aseptic techniques and antibiotics are used in the operation, the host is still extremely vulnerable to microbial infections [6]. The interaction between the implant and the host’s immune system will cause the rejection of foreign bodies, causes inflammation and damage to the tissue surrounding the implant, eventually lead to implantation failure [7]. Therefore, imparting the antibacterial ability to the surface of the implant has become a research hotspot. There are two main types of surface antibacterial functional modification methods for metal implants. One is a chemical method based on the release of metal antibacterial ions, for instance, Ag^+^, Zn^2+^, Cu^2+^, Mg^2+^ [8–10]. The other is a physical method that produces a special surface structure to resist bacterial colonization but supports cell adhesion and proliferation [11, 12]. However, both methods have certain limitations, excessive release of metal ions may cause undesirable toxic and side effects, and the antibacterial bionic structure may be that the effect is not obvious enough [13–15]. Based on the two surface modification methods, a new method that can generate germicidal ions and antibacterial microstructures was proposed, make the surface of the material have photocatalytic and photothermal functions, which has higher antibacterial efficiency and does not damage the surrounding tissue cells within a safe range [16, 17]. Photocatalytic antibacterial refers to the principle that reactive oxygen species (ROS) are produced under visible light or near-infrared (NIR) light to oxidize the phospholipids and proteins of bacteria, thereby deactivating cells [18]. Photothermal is relying on materials to convert light energy into heat and to kill bacteria through local heating. Carbon-based nanomaterials [19], gold nanorods [20], copper nanoparticles [21] and so on are mainly used for photocatalytic and photothermal antibacterial materials in biomedical materials.

In our previous work, a series of new biomedical alloys with high tensile stress, large elongation, low elastic moduli and good superelasticity and shape memory effects were developed, such as Ti-19Zr-10Nb-1Fe (TZNF) and Ti-16Zr-10Nb-4Ta [22–24]. Nanotube arrays were prepared on this series of alloys for photocatalytic water splitting [25] and osteocyte differentiation [26]. Due to the stable physical and chemical properties, non-toxicity and easy availability of TiO_2_ coatings, many studies have been conducted on the biological application of titanium alloys. To further endow TiO_2_ coating with antibacterial properties, innovative development of photocatalytic and photothermal synergistic antibacterial functions is introduced, a nano-bowl-shaped TiO_2_ coating was creatively prepared on TZNF alloy, which can absorb NIR light to antibacterial and promote the proliferation of osteoblasts. Anatase and rutile TiO_2_ has been proven to be used as a photosensitizer in photocatalytic reactions is very common, but TiO_2_ has a wide band-gap, it can absorb ultraviolet light or sunlight [25], and long-term ultraviolet light is harmful to the living. To avoid ultraviolet light irradiation, the preparation of the bowl-shaped TiO_2_ oxide layer uses a template method, using block polymers as a template [27]. The abundant C, N elements of polystyrene-poly(4-vinylpyridine) (PS-P4VP) can sinter residual so that the bowl-shaped TiO_2_ is doped with non-metallic elements to reduce the band-gap [27–29]. The combined effect of ROS generated by photocatalysis on the surface of bowl-shaped TiO_2_ during NIR light irradiation and the increase in local surface temperature makes it produce excellent antibacterial properties around the implant. At the same time, due to the increased hydrophilicity of the bowl-shaped TiO_2_ coating, it can also increase the proliferation and adhesion of osteoblasts, and the short-term irradiation of NIR light will not cause the intolerable high temperature of living beings and the damage of osteoblasts.

## Materials and methods

### Synthesis of polyvinyl pyridine

The chain initiator pentanoic acid and monomer 4-vinyl pyridine were weighed 0.1 g and 3.75 mL, respectively, both added to a 50 mL Schlenk bottle together, and then 5 ml ethanol solvent with 0.01 g azobisisobutyronitrile was added. The Schlenk bottle was put into liquid nitrogen, freeze-thaw, and blow nitrogen gas at the same time, repeated three times to remove the excess air in the bottle. The Schlenk bottle is heated in a 70°C oil bath for 24 h and transferred to liquid nitrogen to quench the reaction. After thawing in a water bath, the solution was settled with petroleum ether, repeated three times and dried at room temperature to obtain polyvinyl pyridine (P4VP). The yield is about 51%.

### Synthesis of PS-P4VP

P4VP and styrene were weighed 0.1 and 0.29 g, respectively, put into 2 mL methanol solvent, and degassed as in the above operation. Then, the bottle was placed in a 70°C oil bath to react for 24 h and also placed in petroleum ether to settle to obtain the final product PS-P4VP. The yield is about 42%.

### Preparation of bowl-shaped surface of TZNF alloy

The TZNF alloy independently developed by our research group was used as the substrate, and the specific preparation method and characterization were published in reference [24]. First, the TZNF alloy matrix was cut into 10 mm × 10 mm × 1 mm pieces, then sand it with sandpaper to remove the surface oxide film and impurities, use ethanol and deionized water to ultrasonically clean and dry for later finally.

PS-P4VP (10 mg) was placed in a 10 mL weighing bottle, 2 mL of tetrahydrofuran solvent was added to fully disperse the polymer in the solution, then ethanol was added slowly, stirred for 2 h, and to make PS-P4VP self-assemble into balls in the tetrahydrofuran/ethanol mixed solvent. The microsphere suspension was placed at room temperature to evaporate the solvent to a final concentration of 2 mg/mL. Titanium (IV) isopropoxide (TTIP) (0.2 mL) was added to this suspension and stirred for 48 h to obtain PS-P4VP microspheres coated with TiO_2_. The product was centrifuged three times repeatedly to remove excess TTIP. These microspheres are filtered by a 450 nm filter membrane for later use. Then the microspheres were uniformly coated on the surface of TZNF alloys by a spin coater (Setcas Electronic, China) and TZNF alloys were placed in a program-controlled electric furnace for calcination. The samples were sintered at 500 °C for 2 h in a nitrogen atmosphere and then sintered at 450 °C for 1 h in air, at a heating rate of 2 °C/min. Finally, the bowl-shaped surface of TZNF alloys was obtained (TZNF-b1). The samples with filtered microspheres coated on the surface of TZNF were denoted as TZNF-b2.

### Characterization

The composition of P4VP and PS-P4VP was confirmed by ^1^H NMR (Bruker ARX 400, Germany). The morphology of samples was observed by field emission scanning electron microscope (SEM) (ZEISS SUPRA55, Germany). The microstructure and composition of the bowl-shaped TiO_2_ coating were detected by field emission transmission electron microscopy (TEM) (JEM-2100Plus, Japan). The surface wettability of samples surface was measured by a surface contact angle goniometer (SL200KS-MD, China) and an image was captured by the camera-equipped instrument.

### Detection of ^1^O_2_ and ·OH and photothermal effects

TZNF and TZNF with the bowl-shaped nanostructures (TZNF-b1 and TZNF-b2) were immersed in 3 mL of 1,3-diphenylisobenzofuran (DPBF) and methyl violet, respectively, for 4 h. Then, the samples were placed under a NIR light (Philips 100 W, Poland) for 30 min, and the concentration of ^1^O_2_ and ·OH groups decomposed in the solution was detected with a UV-spectrophotometer (Thermo Evolution 300, USA) every 10 min. To reduce water evaporation when NIR irradiated, the methyl violet and DPBF solutions were cooled in an ice-water bath. The temperature of the samples was determined by a thermal camera (Fotric 220S, China) at an interval of 5 min.

### Antibacterial properties

*Staphylococcus aureus* (CGMCC1.282) and *Escherichia coli* (CGMCC1.12883) used in antibacterial experiments are from China General Microbiological Culture Collection Center. The components of the bacterial culture medium are 3 g beef extract, 10 g peptone, 5 g NaCl, deionized water 1000 mL, and the pH is adjusted to 7.4 ± 0.2. *Staphylococcusaureus* and *E.coli* were cultured in the bacterial culture medium at 37 °C for 12 h, and then the two bacteria were dispersed in a PBS buffer solution by centrifugation to a concentration of 1 × 10^7^ CFU/mL, i.e. the concentration at which the absorbance of *S.aureus* and *E.coli* were measured with a 600 nm UV-spectrophotometer at 0.5 and 1.0, respectively. Bacterial suspension and the samples were incubated for 2 h, then taken out and placed in another well plate, added 240 μL of PBS solution and NIR light for half an hour. After the treatment, samples were shaken for 5 min, draw 80 μL from each well and apply to the agar plate. After incubation at 37°C for 24 h, the number of colonies on the agar medium was counted and photographed. The antibacterial rate can be calculated by the formula *R* = [(*λ*_0_−*λ_t_*)/*λ*_0_]×100%, in which *R* is the antibacterial rate (%), *λ*_0_ is the number of colonies cultured on TZNF without NIR light and *λ_t_* is the number of colonies co-cultured on the surface of TZNF, TZNF-b1 and TZNF-b2 after illumination.

### Protein adsorption assay

The Bradford Protein Assay Kit (Beyotime, China) is used to determine the amount of protein adsorbed on the surface of samples, and the protein concentration determined by the Bradford method is not affected by most of the chemical substances in the sample. First, dilute the protein standard solution to the standard concentration of 0, 25, 50, 100, 200, 300, 400 and 500 µg/mL, measure the absorbance at 570 nm wavelength with a microplate reader (Biotek Epoch Elx808, USA), and draw according to the positive relationship of concentration–absorbance standard curve line. Second, the samples were soaked in fetal bovine serum albumin for 12 h, respectively, then were taken out and transferred to 200 μL of PBS solution containing 0.1% SDS (surfactant), and shaken for 15 min to measure the absorbance value. According to the protein concentration–absorbance curve, the amount of protein adsorbed on the surface of samples is calculated.

### Cell proliferation and adhesion

Mouse pre-osteoblasts (MC3T3-E1) cells were provided by Peking Union Medical College Hospital. MC3T3-E1 grows in a culture medium containing 10% (volume) fetal bovine serum in DMEM (GE Life Science-Hyclone) medium with 100 U/ml penicillin and 100 mg/l streptomycins. The culture medium was stored in a cell incubator with 37 °C and 5% CO_2_ and changed every 2 d. The samples of TZNF, TZNF-b1 and TZNF-b2 were soaked in ethanol for 2 h and placed under ultraviolet light for light sterilization. Then, the three samples were put in a 24-well plate to co-culture with MC3T3-E1 for 7 d and inject 1 mL of 1 × 10^4^ cell/mL cell suspension into each well. The illuminated samples were taken out and exposed to light for 0.5 h under the NIR lamp every day. The proliferation of the cells co-cultured with the sample was tested on the 1, 3 and 7 d using Cell Counting Kit (CCK)-8 (Beyotime, China). The optical density of the solution at 450 nm was measured by the microplate reader. The samples cultured for 3 d were fixed with 2.5% glutaraldehyde, and then stained with Actin-Tracker-Red and DAPI for 30 and 15 min, then placed under a fluorescence microscope to observe and take photos.

## Results and discussion

**Figure 1.**
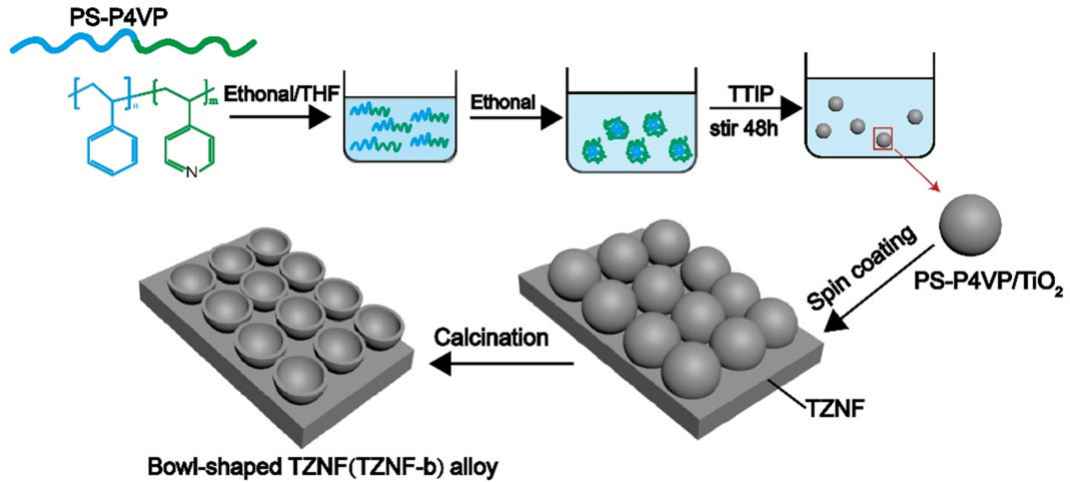
Schematic diagram of preparing bowl-shaped TiO_2_ coating on TZNF alloy

Figure 1 shows schematically the preparation process of the bowl-shaped TiO_2_ coating on TZNF alloy. First, the amphiphilic block copolymer polystyrene-polyvinyl pyridine (PS-P4VP) was prepared through atom transfer radical polymerization reaction and self-assembled into balls in different mixed solvents [30]. The block copolymer PS-P4VP obtains different micelle morphologies in different mixed solvents of tetrahydrofuran/water and tetrahydrofuran/ethanol, adjusting the mixing ratio of the two solvents in the tetrahydrofuran/ethanol system, the morphology of the micelles will also change in size and morphology [31]. The second step is to coat TiO_2_ on the surface of polymer nanoparticles, and obtain a PS-P4VP/TiO_2_ microsphere suspension with controllable morphology by adjusting the hydrolysis rate of TTIP [32, 33]. Third, PS-P4VP/TiO_2_ microspheres were spin-coated on TZNF alloy and sintered in N_2_ and air, respectively, to finally obtain a bowl-shaped coating. The sintering under N_2_ is to keep the bowl-shaped morphology as much as possible and prevent the PS-P4VP/TiO_2_ microspheres from collapsing at high temperatures, while the air sintering is to better remove the PS-P4VP polymer residue. Due to the high electron-hole recombination rate of TiO_2_, the photocatalytic efficiency is low, and the wide band-gap makes the light response range of TiO_2_ lower [34]. PS-P4VP nanoparticles can not only be used as a template for the formation of bowl-shaped TiO_2_ but also introduced rich in C and N elements. Due to the large forbidden band width (3.0∼3.2 eV) of TiO_2_, photocatalytic can only occur under ultraviolet light irradiation, and long-term irradiation of ultraviolet light will cause lesions to the human body, so the doping modification of non-metallic elements TiO_2_ for its high photocatalytic activity under NIR [28, 29]. The surface of the bowl-shaped nanostructure can allow light to be reflected multiple times on the bowel wall, the utilization rate of light is higher, and the photocatalysis and photothermal effects are further improved.

**Figure 2.**
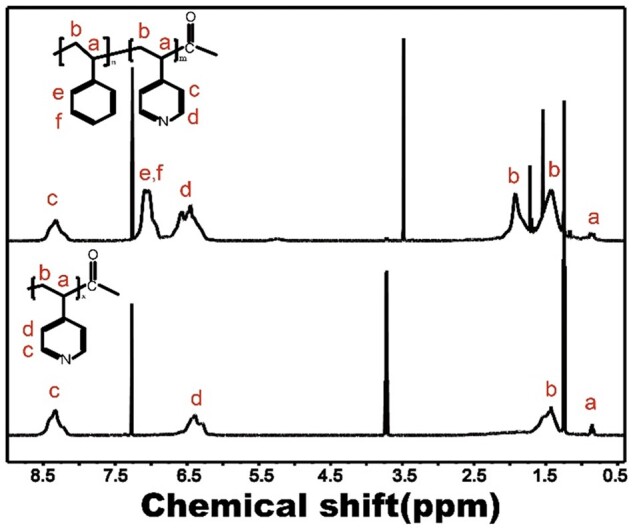
^1^H NMR spectrum of PS-P4VP

The structure of PS-P4VP was characterized by ^1^H NMR spectroscopy, different chemical shifts correspond to hydrogen at different positions to judge the molecular structure of the polymer, as shown in Fig. 2. The characteristic peaks of the methine group on ethylene appear at *δ* =  0.8 (a) and *δ* = 1.5 (b), respectively. *δ* = 8.2 (c) and *δ* = 6.5 (d) are the characteristic peaks of pyridine heterocycle, respectively. After the copolymerization of PS and P4VP, a new peak appears around *δ* = 6.87.1, which is the characteristic peak of benzene. *δ* = 7.2 is the characteristic peak of solvent CDCl_3_. The two sharp peaks at *δ* = 3.7 and 1.2 are the characteristic peaks of the residual solvent ethanol in P4VP. *δ* = 3.5, *δ* = 1.7 and *δ* = 1.5 positions may be residual water peaks in the CDCl_3_.

**Figure 3.**
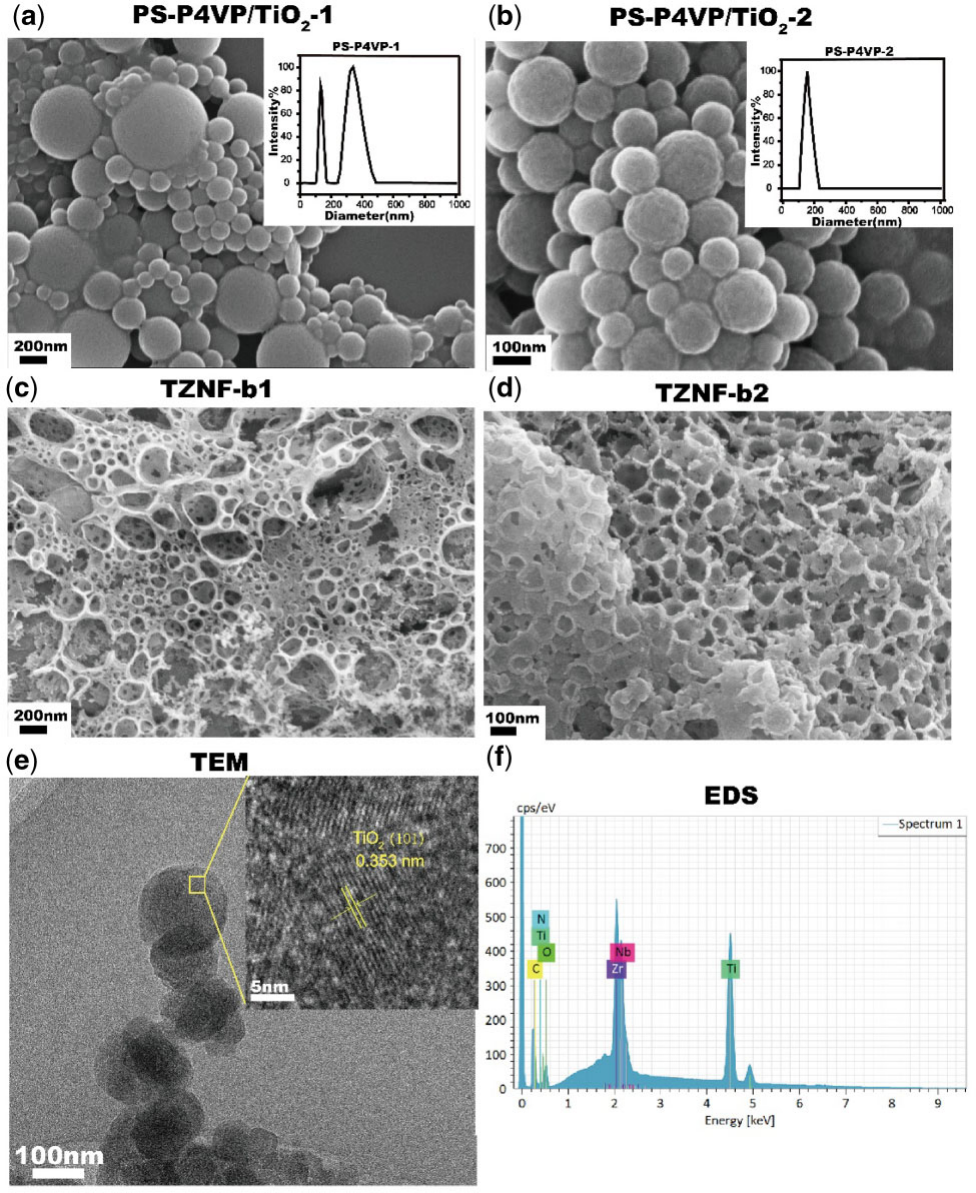
SEM images of PS-P4VP/TiO_2_-1 (**a**), PS-P4VP/TiO_2_-2 (**b**), TZNF-b1 (**c**) and TZNF-b2 (**d**), and the nanoparticle size and distribution chart of PS-P4VP-1 [insert in (a)] and PS-P4VP-2 [insert in (b)]; TEM image (**e**) and EDS image (**f**) of the bowl separated from TZNF-b2

After successfully preparing the PS-P4VP amphiphilic block copolymer, it was measured the size and dispersibility of the self-assembled microspheres. PS-P4VP-1 has a wide particle size distribution directly in ethanol/tetrahydrofuran solution, and the particle sizes are mainly 175 ± 36 nm and 396 ± 52 nm (the inset in Fig. 3a). After filtering with a 450 nm filter membrane, microspheres with a particle size of 182 ± 27 nm are left (PS-P4VP-2) (the inset in Fig. 3b). PS-P4VP polymers of different particle sizes are used as templates, and the sizes of the TiO_2_ shells after coating are different, resulting in different sizes of the bowls finally sintered on the TZNF alloy. The diameter of the bowl on TZNF can be controlled by adjusting the particle size of PS-P4VP. The particle size of the microspheres after TiO_2_ shell coating polymer is consistent with that of the PS-P4VP microspheres, but it is easy to stick (Fig. 3a and b). Compared with TZNF-b1 (Fig. 3c), the morphology of the TZNF-b2 (Fig. 3d) has a uniform and smaller bowl diameter, so it has a larger specific surface area, which provides better conditions for subsequent photocatalysis and cell adhesion. The bowl-shaped structure after surface modification of TZNF was further analyzed by TEM (Fig. 3e). The bowl-shaped surface is composed of C, N-codoped TiO_2_ (Fig. 3f) and there are lattice stripes on the anatase TiO_2_ (101) plane, the lattice spacing is 0.353 nm. The chemical composition of the bowl-shaped TiO_2_ coating and the TZNF matrix is similar, making the two easier to combine after sintering [35].

**Figure 4.**
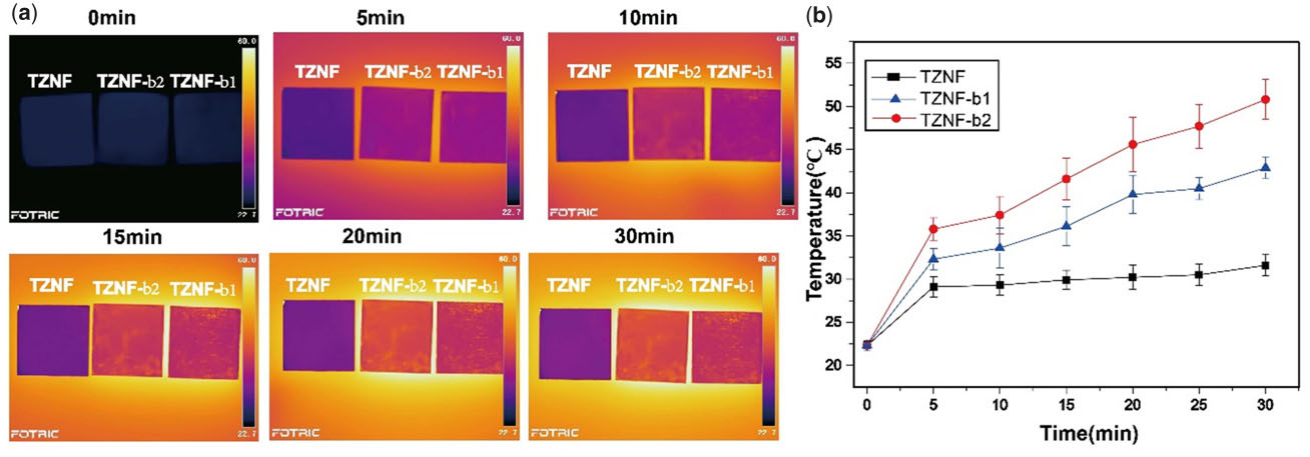
TZNF, TZNF-b1 and TZNF-b2 thermal imaging pictures (**a**) and temperature change diagram (**b**) under NIR irradiation for 30 min. The data are represented as mean ± standard deviation, *n* = 3

NIR irradiation not only produces a photocatalytic reaction but is also accompanied by a thermal effect. The intuitive experience of the surface temperature change of the sample under NIR irradiation within 30 min is shown in Fig. 4a. As shown in Fig. 4b, the temperature of the sample surface will increase with the extension of the irradiation time, but after reaching a certain level, the increase rate decreases, such as TZNF. Compared with TZNF-b1, TZNF-b2 has a higher photothermal conversion efficiency, which is consistent with the result of photocatalysis, indicating that the utilization rate of TZNF-b2 for NIR light indeed exceeds that of TZNF-b1. The surface temperature of TZNF-b2 is about 52°C after 30 min of NIR light, and the temperature of TZNF-b1 is about 43°C. Within 30 min, the surface temperature of TZNF-b1 and TZNF-b2 has not reached the plateau. Generally, as the irradiation time increases, the temperature rises gradually, and the smaller the diameter of the bowl, the higher the conversion efficiency for light and heat, finally reaching a plateau. However, considering the impact of the living body’s tolerance to NIR light and the surface temperature of the implant after the actual implantation, the light time should not be increased. In addition to the optical properties of TiO_2_, the nano-bowl structure also makes a significant contribution to the photothermal effect, nanostructures can improve heat transfer performance and promote light-to-heat conversion [36, 37].

**Figure 5.**
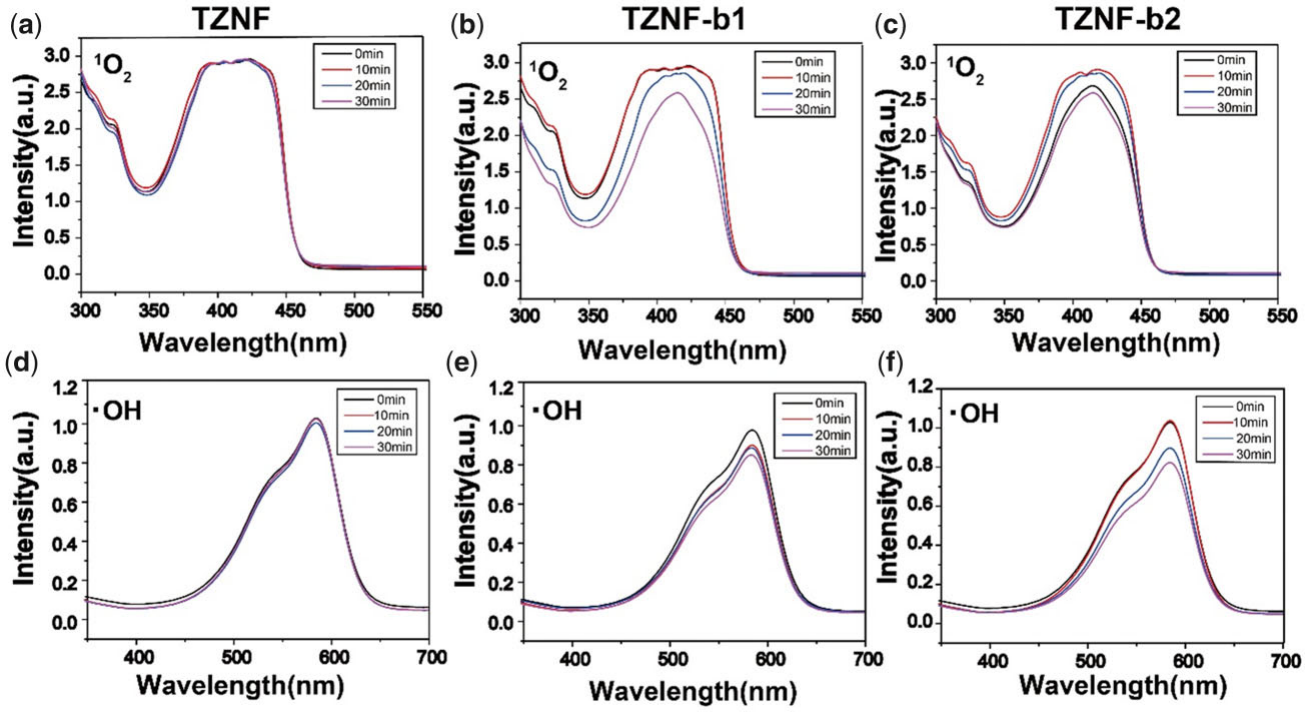
Detection of ROS upon irradiation with NIR light for 30 min: ^1^O_2_ and ·OH detected from the decay of DPBF and methyl violet of TZNF (**a** and **d**), TZNF-b1 (**b** and **e**) and TZNF-b2 (**c** and **f**)

In the experiment of the photocatalytic function of the bowl-shaped surface doped with C, N on TZNF alloy under NIR light, the UV spectrum of the released ROS ion released by TZNF, TZNF-b1 and TZNF-b2 in DPBF and methyl violet is as follows (Fig. 5). DPBF and methyl violet can degrade ^1^O_2_ and ·OH ions, respectively [38]. The decrease in UV absorption intensity at around 420 and 580 nm represents an increase in the released ROS , the better the photocatalytic performance of samples. The TZNF alloy matrix will not release ROS ions under NIR light and has no photocatalytic function (Fig. 5a and d). TZNF-b1 and TZNF-b2 degraded DPBF and methyl violet content more and more over time under NIR light, indicating that the bowl-shaped TiO_2_ modified coating endows the TZNF matrix photocatalytic ability. Compared with TZNF-b1, TZNF-b2 has better photocatalytic performance, because TZNF-b2 degrades 11.1% of DPBF and 20.4% of methyl violet after 30 min of NIR light (Fig. 5c and f), while TZNF-b1 is only 9.6% and 12.4% (Fig. 5b and e). From the time node of 10 and 20 min, the degradation of DPBF and methyl violet by TZNF-b2 is also faster, indicating that TZNF-b2 has a higher light utilization rate for NIR, which is related to the more uniform bowl diameter and comparatively large surface area of TZNF-b2.

**Figure 6.**
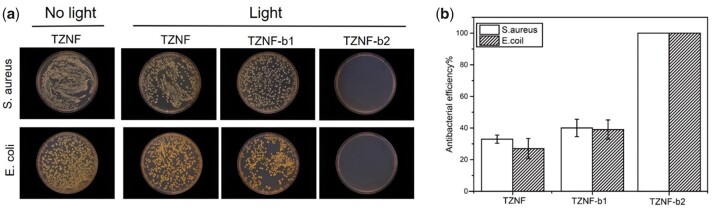
(**a**) Bacteria colony images of TZNF without NIR light and TZNF, TZNF-b1, TZNF-b2 after NIR light; (**b**) antibacterial rates of the TZNF, TZNF-b1 and TZNF-b2 under NIR irradiation, TZNF without NIR light as the control. The data are represented as mean ± standard deviation, *n* = 3

The antibacterial activity of TZNF-b1 and TZNF-b2 against *S.aureus* and *E.coli* was evaluated by the diffusion plate coating method, and the unirradiated TZNF alloy was used as a control. As shown in Fig. 6a, TZNF-b2 has the highest antibacterial rate, and the efficiency of killing *S.aureus* and *E.coli* under the synergistic effect of photocatalysis and photothermal effect is as high as 100%. From the results of colony statistics, after 30 min of light, TZNF, TZNF-b1 and TZNF-b2 all have antibacterial properties, and TZNF with a nano-bowl structure has better antibacterial activity. The TZNF matrix has 32% and 29% antibacterial properties against *S.aureus* and *E.coli* under NIR light, in other words, the single heat effect can destroy about 30% of bacterial activity. Besides the thermal effect, the antibacterial efficiency of TZNF-b1 and TZNF-b2 is significantly higher than that of TZNF, the ROS released by photocatalysis also play an important role in the antibacterial process. The antibacterial activity of TZNF-b1 is much lower than that of TZNF-b2. The antibacterial rate of TZNF-b1 to *S.aureus* and *E.coli* is about 40%, which is only slightly better than TZNF. It can be seen from the trend of antibacterial amount on the surface of different samples that the combined action of high temperature and ROS can effectively sterilize. Compared with TZNF-b1, TZNF-b2 presents a cliff-like sterilization performance. The reason may be that the ROS ions released by TZNF-b1 and the temperature of light and heat did not reach the standard concentration and temperature of sterilization, leading to a lower antibacterial rate [39, 40].

**Figure 7.**
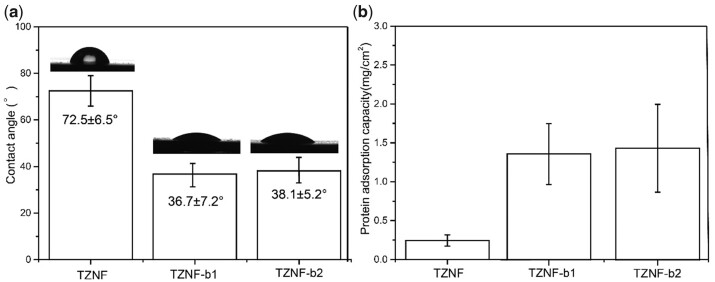
TZNF, TZNF-b1 and TZNF-b2 surface contact angle diagram (**a**) and surface protein adsorption diagram (**b**). The data are represented as mean ± standard deviation, *n* = 6

The surface of hydrophilic biomaterials can promote cell growth and protein adhesion, and cell proliferation and differentiation will be affected by microscopic roughness and wettability, improving the biocompatibility of the material [41, 42]. Figure 7a shows the surface hydrophilicity of TZNF, TZNF-b1 and TZNF-b2. The surface contact angle of TZNF alloy is 72.5 ± 6.5°, and the hydrophilicity of the nano-bowl-shaped TiO_2_ coating is improved after modification, and the contact angle becomes 36.7 ± 7.2° and 38.1 ± 5.2°, the bowl diameter has little effect on the surface contact angle. The increase in surface roughness leads to an increase in hydrophilicity, which greatly improves the protein adsorption on the sample surface. From the statistical graph (Fig. 7b) of protein adsorption on the surface of 1 cm^2^ of TZNF, TZNF-b1 and TZNF-b2, the protein adsorption on the smooth TZNF alloy surface is only 0.246 ± 0.08 mg/cm^2^, while protein content adsorbed on TZNF-b1 and TZNF-b2 is 1.36 ± 0.39 mg/cm^2^ and 1.43 ± 0.56 mg/cm^2^, respectively. There is little difference between the surface contact angle and protein adsorption capacity of TZNF-b1 and TZNF-b2, because the change of the bowl diameter is a nanoscale change, and the test of contact angle and protein adsorption capacity is a macroscopic test of the entire sample surface. The hydrophilicity and protein adsorption capacity of the surface of the nano-patterned sample were significantly improved compared to the pure metal sample surface.

**Figure 8.**
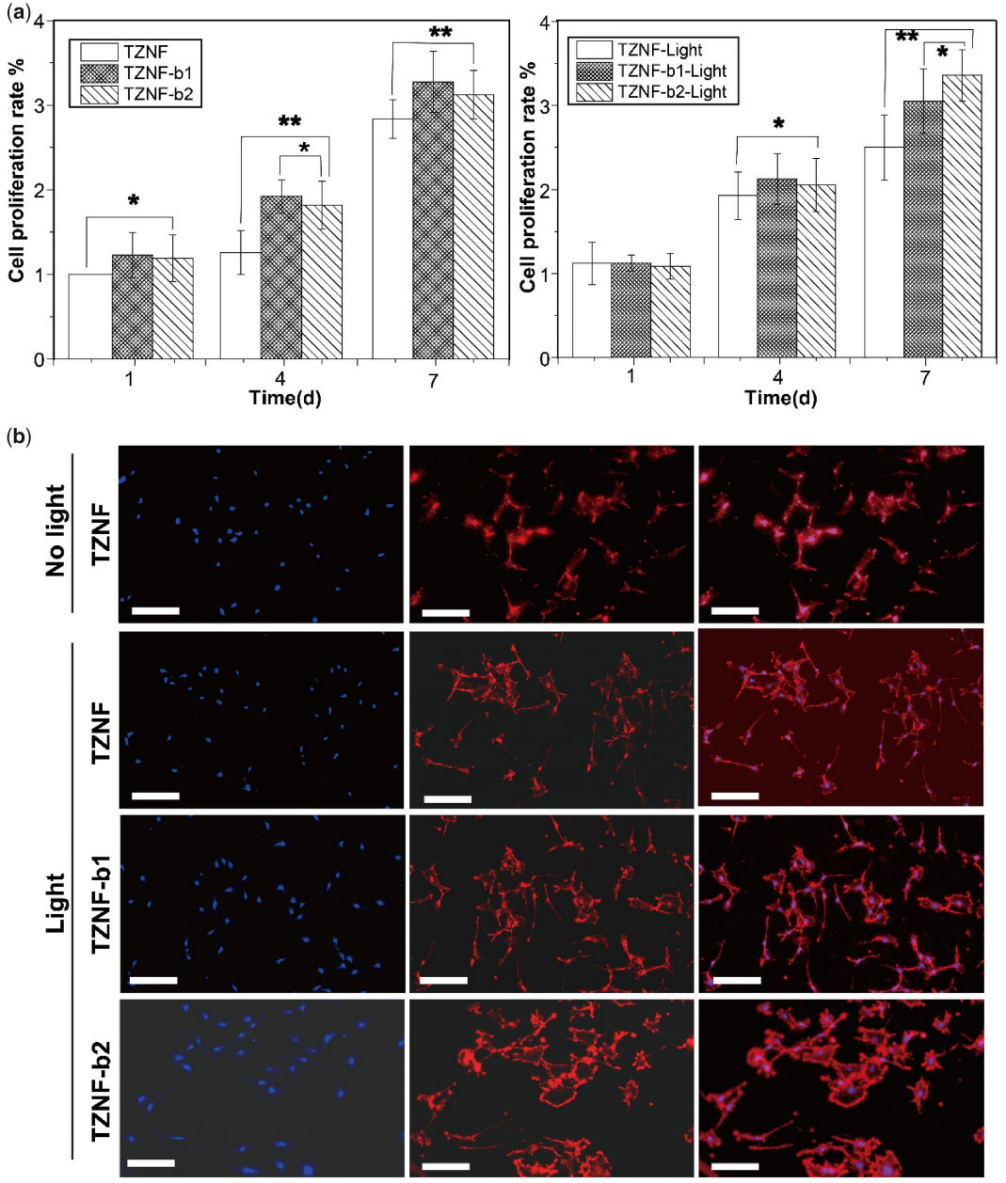
(**a**) Cell proliferation measured by CCK8 after co-culturing with TZNF, TZNF-b1 and TZNF-b2 before and after NIR radiation for 1, 4 and 7 days; (**b**) fluorescence images of MC3T3-E1 culture on TZNF, TZNF-b1 and TZNF-b2 for 3 days. The scale bar is 50 µm. The cytoskeleton was stained by Actin-Tracker-Red (red) and cell nuclei were counterstained by DAPI (blue). TZNF without NIR light as the control. The data are represented as mean ± standard deviation, n = 6 (*P < 0.01, **P < 0.01)

Figure 8a shows the cell proliferation of MC3T3-E1 osteoblasts before and after TZNF, TZNF-b1 and TZNF-b2 NIR irradiation. TZNF-b1 and TZNF-b2 have more cells proliferated from the first day than the TZNF surface (*P* < 0.05), and the amount of cell proliferation increases more overtime after the third day (*P* < 0.01). It has been shown that the nano-patterned surface can improve the adhesion, diffusion and differentiation of osteoblasts, and the underlying mechanism is mediated by the cell arrangement of the cytoskeleton and the formation of adhesion spots. To study the influence of NIR light irradiation, the cells on the samples were irradiated with NIR outside the well plate for 30 min every day. On the third day, the cells were fluorescently stained to observe the morphology of MC3T3-E1 cells and shown in Fig. 8b. The co-cultured cells of the three samples have good morphology and strong cell viability. The osteoblasts on TZNF spread poorly compared with TZNF-b1 and TZNF-b2, while the MC3T3-E1 on the bowl-shaped TiO_2_ coating is polygonal, with a large number of filopodia and lamellipodia, indicating the nano-bowl structure effectively promotes cell proliferation and adhesion. Although thermal effect and ROS produced an inhibitory effect on the bacteria, the cells spread well on the bowl-shaped TiO_2_ coating under the long-term additional culture of osteoblasts, as the result of the different mechanisms of bacteria and cells proliferating on the surface. The attachment of bacteria to the surface of the material and subsequent reproduction depends on the formation of colonies, while mammalian cells can adhere to the surface of implants as single cells mediated by proteins, while nanostructure can already promote the expression of a variety of proteins in cells, thereby promoting the assembly of cytoskeletal structures. In addition, although local overheating is harmful to cells, the growth process of cells is a long-term process, and subsequent growth and proliferation can be gradually repaired [43–45].

## Conclusion

In summary, based on a PS-P4VP polymer template, a nano-bowl-shaped TiO_2_ coating was prepared on TZNF alloy, and a multi-mechanism synergistic antibacterial mode was established under NIR light, and the surface nanostructure promotes the proliferation and adhesion of osteoblasts. The reason why the bowl-shaped TiO_2_ coating is antibacterial is that C, N-codoped TiO_2_ absorbs NIR light and produces photocatalytic and photothermal effects, which yield ROS and heat to inactivate bacteria (both Gram-positive and Gram-negative bacteria). The surface modification of the nano-bowl-shaped structure improves the surface hydrophilicity of the metal matrix and improves the adsorption capacity of the surface protein. Short-term irradiation of NIR light does not affect the growth of osteoblasts but also promotes the proliferation, diffusion and adhesion of osteoblasts. This work demonstrates a practical strategy for endowing implant materials to efficiently antibacterial and promotes cell growth around tissues without the use of antibiotics, thereby reducing potential bacterial-related complications in the actual clinical process. This type of surface modification method for antibacterial materials with multiple antibacterial mechanisms is expected to become a promising method with high biosafety, further expanding the range of options for metal implants.

## Supplementary data 

Supplementary data are available at *REGBIO* online.

## Funding

This work was supported by the National Key Research and Development Program of China (No. 2018YFC1106600) and the National Natural Science Foundation of China (No. 52173193).

*Conflict of interest statement*. The authors declare that they have no known competing financial interests or personal relationships that could have appeared to influence the work reported in this paper. 

## Supplementary Material

rbac025_Supplementary_Data

## References

[1] Konopatsky AS , Dubinskiy SM, Zhukova YS, Sheremetyev V, Brailovski V, Prokoshkin SD, Filonov MR. Ternary Ti-Zr-Nb and quaternary Ti-Zr-Nb-Ta shape memory alloys for biomedical applications: structural features and cyclic mechanical properties. Mater Sci Eng A 2017;702:301–11.

[2] Yu Z-T , Zhang M-H, Tian Y-X, Cheng J, Ma X-Q, Liu H-Y, Wang C. Designation and development of biomedical Ti alloys with finer biomechanical compatibility in long-term surgical implants. Front Mater Sci 2014;8:219–29.

[3] Qu WT , Sun XG, Yuan BF, Li KM, Wang ZG, Li Y. Tribological behavior of biomedical Ti–Zr-based shape memory alloys. Rare Met 2017;36:478–84.

[4] Zhu L , Tang B, Ding M-X, Liu Y, Chen X-F, Yan S-P, Li J-S. Interface characteristic and mechanical performance of TiAl/Ti-2AlNb diffusion bonding joint with pure Ti interlayer. Rare Met 2020;39:1402–62.

[5] Oliveira N , Ferreira EA, Duarte LT, Biaggio SR, Rocha-Filho RC, Bocchi N. Corrosion resistance of anodic oxides on the Ti-50Zr and Ti-13Nb-13Zr alloys. Electrochim Acta 2006;51:2068–75.

[6] Chuang TW , Chen MH, Lin FH. Preparation and surface characterization of HMDI-activated 316L stainless steel for coronary artery stents. J Biomed Mater Res A 2008;85:722–30. 17896759 10.1002/jbm.a.31451

[7] Werner Z , Parham S. Pathogenesis of implant-associated infection: the role of the host. Semin Immunopathol 2011;33:295–306. 21603890 10.1007/s00281-011-0275-7

[8] Lan Z , Guo J, Huang X, Zhang Y, Yong H. Dual function of Cu-doped TiO_2_ coatings on titanium for the application of percutaneous implants. J Mater Chem B 2016;4:3788–800. 32263317 10.1039/c6tb00563b

[9] Li D , Li Y, Shrestha A, Wang S, Wu Q, Li L, Guan C, Wang C, Fu T, Liu W, Huang Y, Ji P, Chen T. Effects of programmed local delivery from a micro/nano-hierarchical surface on titanium implant on infection clearance and osteogenic induction in an infected bone defect. Adv Healthcare Mater 2019;8:e1900002. 10.1002/adhm.201900002 30985090

[10] Xue X , Lu L, He D, Guan Y, Li Y. Antibacterial properties and cytocompatibility of Ti-20Zr-10Nb-4Ta alloy surface with Ag microparticles by laser treatment. Surf Coating Technol 2021;425:127716.

[11] Hasan J , Xu Y, Yarlagadda T, Schuetz M, Spann K, Yarlagadda PK. Antiviral and antibacterial nanostructured surfaces with excellent mechanical properties for hospital applications. ACS Biomater Sci Eng 2020;6:3608–18. 33463169 10.1021/acsbiomaterials.0c00348

[12] Mo S , Mehrjou B, Tang K, Wang H, Huo K, Qasim AM, Wang G, Chu PK. Dimensional-dependent antibacterial behavior on bioactive micro/nano polyetheretherketone (PEEK) arrays. Chem Eng J 2020;392:123736.

[13] Azeredo D , Henriette MC. Antimicrobial nanostructures in food packaging. Trends Food Sci Technol 2013;30:56–69.

[14] Modaresifar K , Azizian S, Ganjian M, Fratila-Apachitei LE, Zadpoor AA. Bactericidal effects of nanopatterns: a systematic review. Acta Biomater 2019;83:29–36. 30273746 10.1016/j.actbio.2018.09.059

[15] Marambio-Jones C , Hoek E. A review of the antibacterial effects of silver nanomaterials and potential implications for human health and the environment. J Nanopart Res 2010;12:1531–51.

[16] Yin M , Li Z, Ju E, Wang Z, Dong K, Ren J, Qu X. Multifunctional upconverting nanoparticles for near-infrared triggered and synergistic antibacterial resistance therapy. Chem Commun (Camb) 2014;50:10488–90. 25068798 10.1039/c4cc04584j

[17] Yuan W , Ji J, Fu J, Shen J. A facile method to construct hybrid multilayered films as a strong and multifunctional antibacterial coating. J Biomed Mater Res B Appl Biomater 2008;85:556–63. 18098202 10.1002/jbm.b.30979

[18] Wang H , Shen J, Cao G, Gai Z, Hong K, Debata PR, Banerjee P, Zhou S. Multifunctional PEG encapsulated Fe_3_O_4_@silver hybrid nanoparticles: antibacterial activity, cell imaging and combined photothermal/chemo-therapy. J Mater Chem B 2013;1:6225–34. 32261695 10.1039/c3tb21055c

[19] Li W , Wang J, Ren J, Qu X. 3D graphene oxide–polymer hydrogel: near-infrared light-triggered active scaffold for reversible cell capture and on-demand release. Adv Mater 2013;25:6737–43. 24123218 10.1002/adma.201302810

[20] Liao J , Shi K, Jia Y, Wu Y, Qian Z. Gold nanorods and nanohydroxyapatite hybrid hydrogel for preventing bone tumor recurrence via postoperative photothermal therapy and bone regeneration promotion. Bioact Mater 2021;6:2221–30. 33553811 10.1016/j.bioactmat.2021.01.006 PMC7829101

[21] Xiaoran D , Kai L, Xuechao C, Bin L, Wei K. A hollow-structured CuS@Cu2S@Au nanohybrid: synergistically enhanced photothermal efficiency and photoswitchable targeting effect for cancer theranostics. Adv Mater 2017;29:1701266. 10.1002/adma.201701266 28745411

[22] Xiong C , Xue P, Sun B, Li Y. Effect of annealing temperature on the microstructure and superelasticity of Ti-19Zr-10Nb-1Fe alloy. Mater Sci Eng A 2017;688:464–9.

[23] Xiong C , Yao L, Yuan B, Qu W, Li Y. Strain induced martensite stabilization and shape memory effect of Ti–20Zr–10Nb–4Ta alloy. Mater Sci Eng A 2016;658:28–32.

[24] Xue P , Li Y, Zhang F, Zhou C. Shape memory effect and phase transformations of Ti–19.5Zr–10Nb–0.5Fe alloy. Scr Mater 2015;101:99–102.

[25] Zhao Y , Zhao K, Yin J, Yang J, Xu J, Gu Y, Liu L, Luo J, Li Y, Sun L. A nanopump for low-temperature and efficient solar water evaporation. J Mater Chem A 2019;7:24311–9.

[26] Wu Y , Li Q, Xu B, Fu H, Li Y. Nano-hydroxyapatite coated TiO_2_ nanotubes on Ti-19Zr-10Nb-1Fe alloy promotes osteogenesis *in vitro*. Colloids Surf B Biointerfaces 2021;207:112019. 34388611 10.1016/j.colsurfb.2021.112019

[27] Li Y , Cao S, Zhang A, Zhang C, Qu T, Zhao Y, Chen A. Carbon and nitrogen co-doped bowl-like Au/TiO_2_ nanostructures with tunable size for enhanced visible-light-driven photocatalysis. Appl Surf Sci 2018;445:350–8.

[28] Asahi RT , Morikawa T, Ohwaki T, Aoki K, Taga Y. Visible-light photocatalysis in nitrogen-doped titanium oxides. Science 2001;293:269–71. 11452117 10.1126/science.1061051

[29] Wang M , Han J, Hu Y, Guo R. Mesoporous C, N-codoped TiO_2_ hybrid shells with enhanced visible light photocatalytic performance. RSC Adv 2017;7:15513–20.

[30] Wang JS , Matyjaszewski K. Controlled/“living” radical polymerization atom transfer radical polymerization in the presence of transition-metal complexes. J Am Chem Soc 1995;28:7901–10.

[31] Cho H , Park H, Park S, Choi H, Huang H, Chang T. Development of various PS-b-P4VP micellar morphologies: fabrication of inorganic nanostructures from micellar templates. J Colloid Interface Sci 2011;356:1–7. 21277585 10.1016/j.jcis.2011.01.001

[32] Sun Z , Kim JH, Zhao Y, Bijarbooneh F, Malgras V, Lee Y, Kang YM, Shi XD. Rational design of 3D dendritic TiO_2_ nanostructures with favorable architectures. J Am Chem Soc 2011;133:19314–7. 22040365 10.1021/ja208468d

[33] Ji BJ , Qiao Z, Dahl M, Lee I, Goebl J, Zaera F, Yin Y. Control of the nanoscale crystallinity in mesoporous TiO2 shells for enhanced photocatalytic activity. Energy Environ Sci 2012;5:6321–7.

[34] Jiang XH , Duan YN, Tian Y, Chen M, Li MK, Liu HH, Yang WL, Tian MK. Facile one-pot hydrothermal method to prepare Sn(II) and N co-doped TiO_2_ photocatalyst for water splitting under visible light irradiation. Rare Met 2022;41:406–9.

[35] Kumar V , Kumar V, Som S, Yousif A, Singh N, Ntwaeaborwa OM, Kapoor A, Swart HC. Effect of annealing on the structural, morphological and photoluminescence properties of ZnO thin films prepared by spin coating. J Colloid Interface Sci 2014;428:8–15. 24910028 10.1016/j.jcis.2014.04.035

[36] Gong X , Gao X, Lei J. Recent progress in bionic condensate microdrop self‐propelling surfaces. Adv Mater 2017;29:1703002. 10.1002/adma.201703002 28845888

[37] Tamás C , Miklós E, Tomi S, Tibor N, Béla H. Simulation of the reflectivity properties of microstructured titanium surface by ray tracing method. J Laser Micro Nanoeng 2015;10:210–5.

[38] Zhang X , Zhang G, Chai M, Yao X, Chen W, Chu PK. Synergistic antibacterial activity of physical-chemical multi-mechanism by TiO_2_ nanorod arrays for safe biofilm eradication on implant. Bioact Mater 2021;6:12–25. 32817910 10.1016/j.bioactmat.2020.07.017 PMC7417618

[39] Hall EK , Singer GA, Kainz MJ, Lennon JT. Evidence for a temperature acclimation mechanism in bacteria: an empirical test of a membrane-mediated trade-off. Funct Ecol 2010;24:898–908.

[40] Torres MA. ROS in biotic interactions. Physiol Plant 2010;138:414–29. 20002601 10.1111/j.1399-3054.2009.01326.x

[41] Callow ME , Fletcher RL. The influence of low surface energy materials on bioadhension-A review. Int Biodeter Biodegr 1994;34:333–48.

[42] Gittens RA , Scheideler L, Rupp F, Hyzy SL, Geis-Gerstorfer J, Schwartz Z, Boyan BD. A review on the wettability of dental implant surfaces II: biological and clinical aspects. Acta Biomater 2014;10:2907–18. 24709541 10.1016/j.actbio.2014.03.032 PMC4103435

[43] Gittens RA , McLachlan T, Olivares-Navarrete R, Cai Y, Berner S, Tannenbaum R, Schwartz Z, Sandhage KH, Boyan BD. The effects of combined micron-/submicron-scale surface roughness and nanoscale features on cell proliferation and differentiation. Biomaterials 2011;32:3395–403. 21310480 10.1016/j.biomaterials.2011.01.029 PMC3350795

[44] Kong Y , Ma B, Liu F, Chen D, Zhang S, Duan J, Huang Y, Sang Y, Wang J, Li D, Liu H, Wang S. Cellular stemness maintenance of human adipose-derived stem cells on ZnO nanorod arrays. Small 2019;15:e1904099. 31738003 10.1002/smll.201904099

[45] Saha S , Pramanik K, Biswas A. Silk fibroin coated TiO_2_ nanotubes for improved osteogenic property of Ti6Al4V bone implants. Mater Sci Eng C Mater Biol Appl 2019;105:109982. 31546427 10.1016/j.msec.2019.109982

