# Supplementary material for: Synergistic antibacterial photocatalytic and photothermal properties over bowl-shaped TiO2 nanostructures on Ti-19Zr-10Nb-1Fe alloy
Source: Regen Biomater. 2022 May 4;9:rbac025. doi: 10.1093/rb/rbac025 (PMC9113230; doi:10.1093/rb/rbac025)
Supplement: rbac025_Supplementary_Data [file rbac025_supplementary_data.docx]

**Support information**


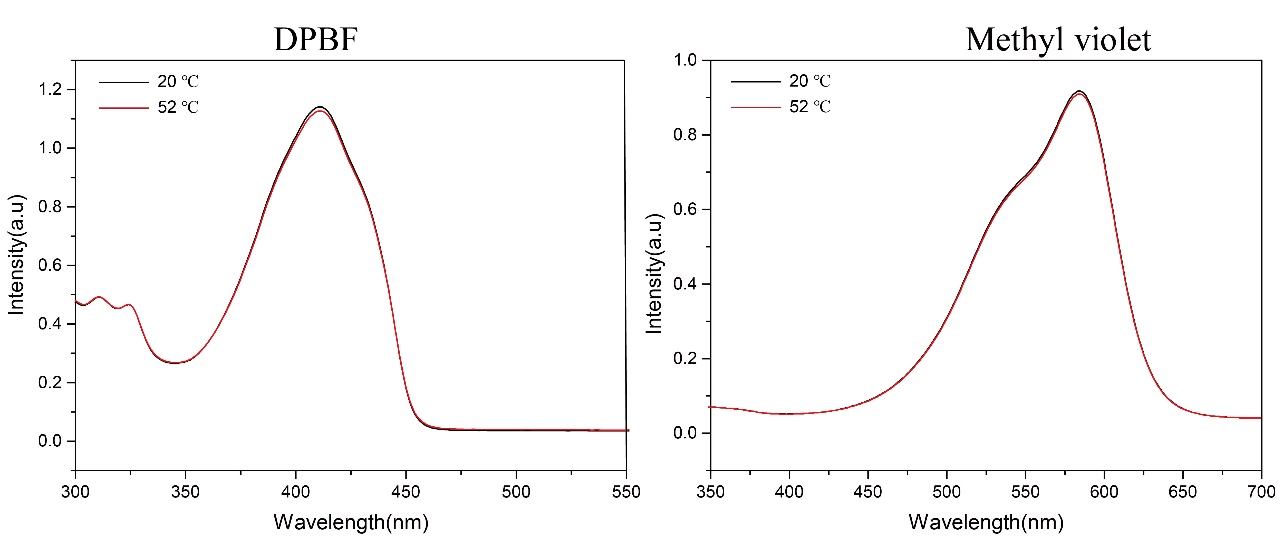


Fig.1^*^ UV spectrophotometer spectra of DPBF and methyl violet of TZNF-b1 at 20 °C and 52 °C for 30 min
